# Female physicians: trends and likely impacts on healthcare in Israel

**DOI:** 10.1186/2045-4015-2-37

**Published:** 2013-09-17

**Authors:** Ziona Haklai, Yael Applbaum, Orna Tal, Myriam Aburbeh, Nehama F Goldberger

**Affiliations:** 1 Division of Health Information, Ministry of Health, 39 Yirmiyahu Street, Jerusalem, Israel; 2 Deputy director, Assaf Harofeh Medical Center, Beer Yaacov, Israel

**Keywords:** Female physicians, Female specialties, Medical workforce, Medical work hours

## Abstract

**Background:**

Female physicians have become an increasing proportion of the medical workforce in Israel. This study investigates this trend and discusses its likely impact on the quantity and quality of medical care available.

**Method:**

Data on licensed physicians and new licenses issued to physicians were taken from a Ministry of Health database, and analyzed by gender, age, academic origin (Israeli graduates, immigrants, Israeli-born who studied abroad), and specialty for the years 1999–2011.

Data on employed physicians, their population group, and work hours were taken from the Central Bureau of Statistics (CBS) annual Labour Force Survey for the years 2009–2011.

**Results:**

The proportion of women amongst physicians aged under 65 rose from 38% in 1999 to 42% in 2011, and was even higher for younger physicians. The highest proportion of females is found amongst new immigrant physicians who studied abroad. The corresponding proportion has been rising steadily amongst Israeli-educated physicians, and is lowest amongst Israeli-born physicians who studied abroad.

Similarly, among newly licensed physicians, the proportion of females has traditionally been highest among immigrants who studied abroad and lowest among Israeli-born graduates who studied abroad. Among newly-licensed physicians who studied in Israel, the proportion of females has historically been intermediate between the other two groups, but it has recently risen to 54% and now parallels the proportion of females among immigrants who studied abroad. In recent years, the mix of academic origins among newly licensed physicians has changed dramatically, with important implications for the proportion of women among newly licensed physicians.

The highest percentage of females was found in family medicine followed by oncology, pediatrics and psychiatry. The greatest increase over the years in this percentage was for gynecology and internal medicine.

Female physicians worked shorter hours than males, particularly at younger ages. The proportion of females among employed Arab physicians is much lower than among Jewish physicians.

**Conclusions:**

The proportion of female physicians has been steadily rising, although in recent years the increase has leveled off. This has been due, in part, to the decline in the flow of immigrant physicians and the increase in the number of Israelis studying abroad. Future developments in medical education options and immigration will determine whether their proportion will continue rising. Planning for future medical personnel must take these results into consideration.

## Background

Comparative international data shows an increase in the proportion of female physicians in all OECD countries. In 17 countries for which data are available for 2000 and 2010, the median proportion of females rose from 35% in 2000 to 41% in 2010, and for physicians under 45, the median proportion was more than half (55%) in 2010 compared with 43% in 2000 [1].

Observation of medical school populations around the world indicates that the number of female physicians will rise even more in the future. In the USA, the proportion of female graduates of medical schools rose from 27% in 1983 to 48% in 2011 [2]. In Canada, the proportion of female medical students has quadrupled in the last four decades from 13% in 1966/7 to 58% in 2006/7 [3]. A recent report of the Royal College of Physicians shows the proportion in the UK to have stabilized at about 57-58% [4].

The latter two studies both show that women favour certain specialties. For example, in Canada, where 32% of all physicians were women in 2007, the leading specialties were pediatrics (48%), gynecology (42%) and psychiatry (37%) [3]. The Royal College of Physicians reported that mapping consultant preferences according to medical fields in the UK revealed that on the axis of people-oriented vs. technology-oriented medical fields combined with “plannable” vs. unpredictable jobs, female physicians prefer people-oriented-“plannable” specialties, such as pediatrics, general practice, psychiatry and public health over surgery, anesthesia and emergency medicine [4].

Data on the number of residency applicants of US medical school graduates in 2012 shows that females accounted for 43% of all applicants, 83% of obstetrics and gynecology applicants, 71% of pediatric applicants, 60% of dermatology applicants and 55% of family medicine applicants [5].

Bowman et al. [6] point out that women tend to choose specialties with lower prestige and lower income and those that involve shorter periods of training. They raise the question of whether the specialties that women choose are more attractive to women because women are less concerned with prestige and money, or because of the effect of subtle discrimination. It may also be that these specialties carry less prestige as a result of the large proportion of women working in them.

The feminization of medicine may lead to decreased productivity as a result of several factors. Women have been found to work shorter hours in various OECD countries [7] and Weizblit at al also found fewer average working hours spent on direct care by female physicians than males in most specialties [3]. Female physicians take more vacation as parents, are more likely to leave the practice of medicine or practice less during child-bearing age, are more likely to retire early and have a higher tendency to work part time [3,6]. Nevertheless, the Royal College of Physicians reported that the great majority of NHS physicians of both genders work full time, and only about 15% have part time contracts. This proportion differs between the hospital sector (8% of men and 21% of women work part-time) and consultants (30% of women consultants have part-time contracts) [4]. However, it should also be noted that work hours are not always correlated with time and care given to patients, since these also depend on quality of work time, and can also vary with time spent on research or administrative tasks.

In Israel, new licenses are issued to graduates of Israeli medical schools associated with five universities who have successfully completed their licensing exams and fulfilled all the requirements of their medical schools, and to those who studied abroad, both immigrants and Israeli-born, who must pass an Israeli licensing examination and provide transcripts from their respective medical schools. These newly licensed physicians can practice in the community in primary care or in hospitals. After completing a one year internship, licensed physicians can then go on to residencies to specialize in their field of interest.

Specialist licenses, granted on completing these residencies and passing specialist exams, are issued for all kind of medical specializations, including for specialists in family medicine, who work in primary care and for pediatricians, who work both in primary care and in hospitals. Specialists are available both at the community level, in health plan clinics and as private consultants, and in hospitals.

The influx of immigrants to Israel from the former USSR in the 1990’s, led to a large increase in the proportion of female physicians in the country [8]. For example, in 1992, a year in which 86% of new licenses were issued to immigrants from Eastern Europe, 56% of these new immigrant physicians were female, and this proportion reached 60% in the following years.

This study focused on the most recent decade, 1999–2011, when immigration was low following the earlier large wave of USSR immigrants. We looked at the composition of the medical workforce over the years, investigating whether the trend of feminization of medicine was continuing, and its relationship with academic origin, immigrant status, and age group. This was computed both for all physicians and for all specialists, to see to what extent females were continuing their studies to achieve specialist status. We also looked into which specialties have experienced the most pronounced rise in the proportion of females and the proportion of females amongst new licenses issued, according to academic origin, as this can help explain our findings, and project future trends.

In addition, we compared the average work hours for male and female physicians in recent years, and the gender of employed physicians by nationality. We discuss the impact of these trends and their sources on quantity and quality of medical care available, and what possible strategies could be taken in order to prepare for the change of balance between the genders of practicing physicians.

## Methods

This study is based on the Physicians License Registry maintained by the Ministry of Health, which includes all physicians and specialists who received a license after graduating in Israel, and for those who graduated abroad, after their qualifications are recognized. The demographic information is periodically updated from the Population Registry at the Ministry of Interior. We focused on physicians aged under 65, which was the retirement age for men until a few years ago. The proportion of female physicians was calculated for the years 1999–2011 by age group (under 45, 45–54, 55–64) and by their academic origin, (country of initial medical studies and birth) grouped into Israeli graduates, Israeli–born who graduated abroad and foreign-born (immigrants) who graduated abroad. This was done for all physicians (specialists and non-specialists together), all specialists and for selected individual specialties: pediatrics, family medicine, gynecology, internal medicine, psychiatry, general surgery, orthopedics, anesthesia and oncology.

Multivariate logistic regression analysis was run to determine the likelihood of licensed physicians aged under 65 in 2011 being female, with year of license (or specialist license for specialist groups) and academic origin serving as control variables. Results are presented in Additional file 1.

Data on employed physicians, their nationality, hours of work in the last week for those employed, and numbers of full time equivalent (FTE) physicians were calculated from the Central Bureau of Statistics (CBS) annual Labour Force Survey for the years 2009–2011 [9]. Number of FTE physicians was based on reported hours of work in the last week and a full time work week of 45 hours.

## Results

### Percentage of females by year and age group

According to the Physicians License Registry in the Ministry of Health, there were 26,065 licensed physicians registered in Israel in 2011 aged under 65 (about 15 thousand males and 11 thousand females), of whom 13,771 also had licenses as specialists [8]. About a third of the physicians were in each age group (35.8% under 45, 30.9% aged 45–54, and 33.3% aged 55–64). Their academic origin was 39.5% Israeli graduates, 12.7% Israeli-born who graduated abroad and 47.8% immigrants who graduated abroad.

Table 1 shows the percentage of females among all physicians and all specialists under 65 by age groups, and among new licenses issued. The years shown are 1989, before the USSR immigration, in 1995 after the large wave of USSR immigrants, and between 1999 and 2011. The percentage of women rose steadily among all physicians, and among the younger age groups, under 45 and 45–54, between 1999 and 2011. For specialists, the increase was even steeper, with an increase of over 60% in the percentage of female specialists in the 45–54 year old age group between 1999 and 2011. In the last three years, 2009–2011, half of all specialists under age of 45 were females compared with 37% in 1999.

**Table 1:** Percentage of females among physicians and specialists at end of each year by year and age, and among new licenses by year of issue

	**Age group/year**	**1989**	**1995**	**1999**	**2001**	**2003**	**2005**	**2007**	**2009**	**2010**	**2011**
**Physicians**	**All ages**	30.0	33.3	38.3	39.0	39.5	39.9	40.5	41.0	41.4	41.8
	**under 45**	27.5	36.4	42.7	44.1	45.4	46.4	47.0	47.2	47.1	47.2
	**45–54**	29.6	26.7	33.2	35.1	36.6	38.0	39.9	41.1	42.0	42.2
	**55–64**	38.8	36.2	36.9	34.4	33.2	32.5	32.7	34.1	34.7	35.7
	**New licenses**	30.1	47.0	47.7	53.3	48.4	43.8	48.5	43.9	44.9	43.1
**Specialists**	**All ages**	25.0	26.2	28.7	30.7	32.2	33.7	35.4	37.0	37.8	38.5
	**under 45**	23.1	30.3	37.1	40.6	43.2	46.3	48.4	50.2	50.6	51.2
	**45–54**	23.0	20.4	23.5	26.5	29.3	31.9	35.3	37.8	39.0	39.5
	**55–64**	31.3	28.6	24.1	21.7	21.0	21.7	22.5	24.4	25.7	27.1
	**New licenses**	23.4	34.0	37.5	41.8	40.9	47.7	51.0	48.9	45.2	43.7

The source of this increase in the percentage of females among all physicians and particularly young physicians is shown clearly by the rising percentage of new physician licenses issued to females. In particular for specialists, the percentage more than doubled between 1989, when 23% of new specialist licenses were issued to females and 2007 when it reached 51%. For all physicians, about a half of the new licenses were issued to females since the end of the 90’s compared to 30% in 1989. However, the trend of an increasing percentage of female new licenses seems to have peaked, and in recent years, 2009–2011 the percentage was somewhat lower.

### Percentage of females by year and academic origin

**Figure 1:**
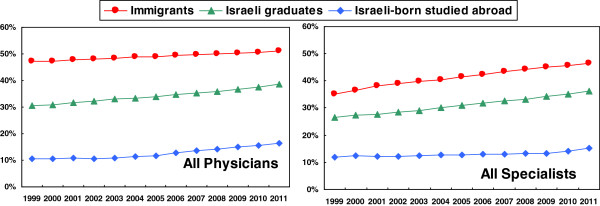
Percentage of females among physicians and specialists under 65 by year and academic origin.

The percentage of females amongst all physicians and all specialists by year and academic origin is shown in Figure 1. The percentage is highest amongst immigrants who graduated abroad, but increased only slightly from 47% in 1999 to 51% in 2011, for all physicians. The percentage rose more, however, for all specialists in this group, from 35% to 46%, probably reflecting those new immigrant physicians who have trained as specialists after coming to Israel. The lowest percentage is amongst Israeli-born physicians who graduated abroad, which had been stable at about 11% until 2004 for all physicians, and then increased to 16% by 2011. The greatest change is for those who graduated in Israel, with a steady increase, from 30% in 1999 to 39% in 2011 for all physicians, and from 27% in 1999 to 36% in 2011 for all specialists.

### Proportion of females by specialty

**Figure 2:**
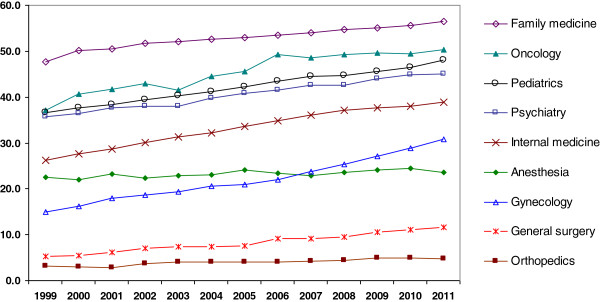
Percentage of females among different specialists under 65 by year.

Figure 2 shows the proportion of females in different specialties from 1999 to 2011. The percentage of females in most specialties has increased, except for anesthesia. The highest percentage of females is in family medicine followed by oncology, pediatrics and psychiatry. The highest increase over the years was for gynecology and internal medicine. Even in general surgery, with low numbers of females, there was an increase between 1999 and 2011. Orthopedics, however, remains a specialty with very few females.

### Percentage of new licenses for females by year and academic origin

To investigate and understand the recent trends in feminization more clearly we need to look further at the breakdown of new licenses by academic origin (country of initial medical studies and of birth) and its changes over time.

Table 2 shows the breakdown of total new licenses issued between 1999 and 2011 by academic origin. We see that between 1999 and 2003, licenses issued to new immigrants who graduated abroad formed over half of new licenses, but their contribution decreased to about a quarter between 2008–2011. Correspondingly, the proportion of new licenses rose amongst the other groups. Between 2005 and 2010 about half of all new licenses issued in Israel were to graduates of Israeli universities instead of a third in 1999–2002. During this period, there was also an increase in the percentage of new licenses that were issued to Israelis who studied abroad, most of them in Hungary, Italy, Romania and Jordan [8]; by 2011 this group accounted for a third (32%) of all new licenses.

**Table 2:** New physician licenses by academic origin, 1999-2011

**Year**	**Number of new licenses issued**	**Israeli graduates**	**Immigrants who graduated abroad**	**Israeli-born who graduated abroad**
**1999**	915	31.3%	59.7%	9.1%
**2000**	897	33.4%	54.6%	11.9%
**2001**	856	33.2%	57.2%	9.6%
**2002**	850	34.5%	56.4%	9.2%
**2003**	814	39.4%	52.3%	8.2%
**2004**	698	43.3%	45.3%	11.5%
**2005**	601	50.6%	33.8%	15.6%
**2006**	584	51.2%	28.4%	20.4%
**2007**	548	56.6%	27.4%	16.1%
**2008**	616	52.3%	24.4%	23.4%
**2009**	776	46.4%	24.1%	29.5%
**2010**	726	48.1%	28.7%	23.3%
**2011**	898	41.1%	26.7%	32.2%

**Figure 3:**
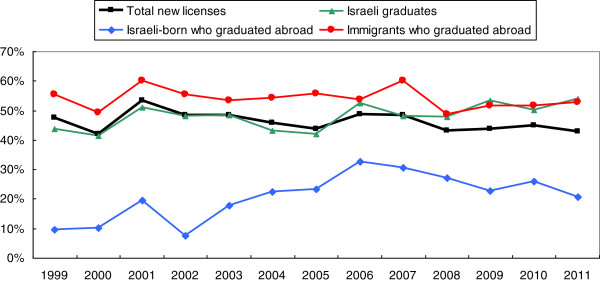
Percentage of females among new physician licenses by year and academic origin.

Figure 3 shows the percentage of females amongst new licenses in these groups between 1999 and 2011. The percentage of females amongst graduates of Israeli universities has increased to about half in the last decade and reached 54% in 2011. Amongst immigrants the percentage is still higher, although in recent years it has been similar to that of Israeli graduates. But amongst Israelis studying abroad, the percentage is much lower - between 21% and 27% in 2008–2011. This has led to the slight decrease in the last 4 years, 2008–2011, in the percentage of females amongst total new physician licenses.

Additional file 1 gives the results from the multiple logistic regression which shows the independent effects of license year and academic origin together in determining the likelihood of physicians being female for the physician workforce aged under 65 in 2011, and for subgroups of different specialties.

### Practicing physicians

Are there differences in the proportion of females between employed and licensed physicians? According to the CBS labour force survey, there were between 23 and 27 thousand physicians employed per year in Israel in 2009–2011, 36% aged under 45, 29% aged 45–54, 27% aged 55–64 and 8% aged 65 and over. Two fifths (40%) of female physicians were under 45 years of age and 5% were 65 and over compared to 33% and 10% of male physicians, respectively.

Only 13% of Israeli Arab physicians were female in 2009–2011, compared to 45% of Jewish and Other physicians. However, the proportion is rising slightly for Arabs, as in 2002–2004 the proportions were 8% and 47% respectively.

Table 3 shows the characteristics of practicing physicians in 2009–2011. The proportion of females among the practicing physicians was 43% in 2009–2011. The proportion of females is greater at younger ages, about half (47%) of physicians under 45 were female compared to two fifths of those aged 45–54 (41%) and 55–64 (43%) and 28% of those aged 65 and over in 2009–2011.

**Table 3:** Characteristics of practicing physicians, 2009-2011

**Age group**	**% female**	**Average work hours/week**		
		**Male**	**Female**	**Ratio male/female**
**Under 45**	47.0	52.7	36.6	1.44
**45–54**	41.3	47.7	38.6	1.24
**55–64**	42.6	48.2	39.6	1.22
**65+**	27.9	30.7	23.2	1.32
**Total**	42.6	47.9	37.3	1.28

Based on CBS labour force survey.

Male physicians worked more hours per week than females, on average 47.9 hours compared to 37.3 for females (male/female ratio = 1.28) in 2009–2011. However the ratio varied with age. Male physicians under 45 worked on average 44% more hours than their female colleagues while those aged 45–64 worked only 22–24% more hours. For those aged 65 and over males worked 32% more hours on average.

Therefore, when numbers of FTE (full time equivalent) physicians, which takes work hours into account, was calculated by gender, we found a total of 15,400 male and 8,900 female practicing physicians on average per year for the years 2009–2011 and the proportion of females was lower, 37%, than their head counts (43%).

In summary, the proportion of females amongst physicians increased between the years 1999–2011, particularly amongst younger physicians, and for the specialties of gynecology and internal medicine. The highest proportion of females was found among immigrant physicians who studied abroad, but has been rising steadily amongst Israeli educated. The proportion was lowest amongst Israeli-born physicians who studied abroad. Similarly, for new licenses issued the highest proportion of females was amongst immigrants who studied abroad but the female proportion has risen sharply amongst Israeli graduates, reaching that of the immigrants. It was lowest amongst Israeli-born who graduated abroad. The numbers of both Israeli graduates and Israeli born graduating abroad have increased in recent years, while the number of immigrant new licenses has been decreasing.

## Discussion

The first aim of this study was to assess the number of female physicians in Israel and to compare the local trend with the trends from other countries. The second objective was to try to understand what impact this may have on the Israeli medical system and what possible strategies could be taken in order to prepare for the change of balance between the genders of practicing physicians.

### Will the feminization of medicine in Israel continue?

Israeli data showed the same trends of increasing feminization of medicine in most specialties, as in other countries. Favored specialties of family medicine, pediatrics, psychiatry and internal medicine are also similar, with orthopedics and general surgery being least popular, reflecting the preference for people-oriented and more “plannable” specialties and in the case of orthopedics, also the high physical strength required. The proportion of females among gynecologists has increased sharply, perhaps reflecting the preference for them among the female patients and therefore the greater opportunities for female physicians to establish flourishing practices in this field.

The group with the highest proportion of female physicians today is immigrants to Israel who studied medicine abroad, a relatively stable proportion over the years, but fewer new licenses have been issued to them in recent years. The steepest increase is among those who studied in Israel. The Committee for Planning Medical Manpower in Israel issued recommendations in their final report in June 2010, which are being implemented and include increasing the numbers of medical students in existing schools in Israel, and opening a new medical school in the Galilee [10]. This can be expected to increase the proportion of Israeli graduates amongst new physician licenses. If the trend of recent years continues of a large proportion of females amongst these Israeli graduates (which reached 54% in 2011, similar to the 57% reported in 2007 in the UK [4]), it will contribute to maintaining the feminization of the physician workforce.

The many new young female doctors were reflected in our finding that almost half (47%) of employed physicians under 45 were female in 2009–2011 according to the CBS labour force survey. The logistic regression results presented in Additional file 1 also showed increasing chance of female physicians by license year while controlling for academic origin, although the trend appears to have passed its peak by 2011 for all physicians, all specialists and certain specialties.

However, we also saw an increasing proportion of new licenses issued to Israeli-born physicians who graduated abroad, amongst whom there is a considerably lower proportion of females.

Who are these graduates and why are they studying abroad? The number of places in medical schools in Israel has been very limited, and did not increase during the decade of 1998–2007. This led to a highly competitive acceptance system. Therefore, many Israelis who wanted to study medicine turned instead to medical schools abroad. In 2011, the largest number of these licenses were issued to graduates of Hungary (83) as well as Romania (50) and Italy (43). In addition, there has been an increasing number of licenses issued in recent years to Israeli-born physicians who studied in Jordan, Egypt or Syria, with 66 new licenses issued to graduates from there in 2011 [8] compared to 6 in 2005.

Why are there fewer females in this group? It could be that females prefer to remain near to home or because they are less comfortable studying in foreign countries. In addition, those who go abroad to study in Arab countries are most likely to be Israeli Arabs amongst whom there is a much higher proportion of male physicians, as we found from the CBS Labour Force Survey.

The lower number of females amongst Israeli-born physicians who studied abroad may have offset some of the increase in the proportion of females among Israeli graduates. This could explain why our data showed that the proportion of females receiving new physician licenses peaked in 2001 and has been lower since then, and that the corresponding proportion for specialist licenses peaked in 2007 and has also been decreasing since then. The proportion of female physicians under 45 has been stable at 47% since 2007. This also may explain why in comparison to OECD countries, in 2000 Israel had a higher proportion of female doctors than the OECD median, while Israel was lower than the OECD median in 2010, showing that the increase in Israel has not kept pace with other countries.

It is hard to know whether the feminization of medical practice in Israel will increase further in future years. On the one hand, the increasing number of Israeli graduates with a high proportion of females would lead to an increase, but the contribution of Israeli-born studying abroad, whose numbers may continue growing, could lead to lower numbers of females. But it could be that with the increasing opportunities to study in Israel, fewer Israelis will opt for the usually more expensive option of studying abroad, and being far from home. The interplay between these factors, as well as future immigration of physicians, will determine whether feminization continues.

### The effect of the work practices of female physicians on the physician workforce

Our data on average work hours showed that male physicians worked more hours per week, 47.9 hours compared to 37.3 for females in 2009–2011 compared to 53.8 and 47.5, respectively, in Canada [3]. The contribution of young female physicians to the medical workforce in the short term will be less (in terms of hours worked) than their numbers, as until age 45 they worked 44% fewer hours than men. However, in the long term, as they become older, after their children grow up, it can be expected that their work hours will increase, as the difference in work hours with their male colleagues is only about 23% at age 45–64. The specialties that women tend to choose contribute to their shorter hours, as family medicine and pediatrics are mostly ambulatory professions with flexible hours and individual work contracts.

Therefore, the impact of the growing proportion of women in medicine may reduce the increase in the medical workforce due to new graduates. The number of FTE additions from new graduates may be less than implied by the number of new physicians, per se. In view of the expected shortage of physician manpower, it is all the more important to try and increase numbers of new physicians, maybe by a further increase in numbers enrolled in medical schools in Israel, and as the Committee recommended, encouraging immigration of physicians and return to Israel of those who study abroad either for their primary or specialist licenses [10].

However, an Israeli study by Shye found that female physicians devote more of their time to patient care. She found that in Israel, as opposed to other countries, females saw more patients per hour than their male colleagues. She also found that women physicians in Israel spent a larger proportion of their total work in direct patient care than males [11]. These two unique facts point to women’s higher productivity in patient care which may offset their shorter hours. However, this is not always recognized because the prestige of a medical department today lies mostly in its research capabilities and the number of scientific publications rather than in patient care quality and efficiency. Therefore, many medical departments will still prefer accepting male residents in expectation of more academic accomplishments. This might tend to discriminate against women in their career advancement.

Female physicians, in particular younger ones, are more likely to work in one place only. In 2010, 29% of female physicians worked in more than one work place, compared to 42% of males, although the proportion is higher for ages 45–54, 37% and 50%, respectively, based on data received from all HMOs and most hospitals [8]. It could be that female work hours could be increased by enabling work in more than one place more easily perhaps by relocating HMO clinics closer to hospitals to make it more feasible for hospital- based female physicians to take on extra positions in ambulatory settings.

### Effects on the organization of the medical workforce

The effect of feminization on the organization of medical practice and professional status are discussed by Ross [12]. She argues that although the feminization of medicine has somewhat lowered the status, influence and salary of physicians, it has also bought welcome changes such as more flexible residency training, more part-time work, maternity and paternity leave, and tax benefits for daycare costs. When a controllable lifestyle has been shown to be an important factor in the choice of specialties by young physicians of both genders [13], these changes are positive and important for the medical profession, and must be encouraged in Israel.

Should government approach workforce planning through policies and regulations, or through market forces? The shortage of manpower in certain specialties recently has drawn attention to the urgency of creating a framework that will allow planned development of the workforce in medicine. Developing incentive systems that will support these goals would be a logical operative to reach the goal.

In Israel, a shortage of specialists has been recognized or expected in the following fields: anesthesiology, general and pediatric surgery, intensive care and pediatric intensive care, neonatology, pediatric psychiatry, pediatric neurology, geriatrics and rehabilitation [10]. Relocation of physicians to countries where salaries are more gratifying poses a threat as well.

Recently, financial incentives have been introduced to encourage physicians to practice in peripheral regions, and to prefer certain specialties that are in shortage, such as anesthesia. Many of the fields with shortages are predominantly male. Are these incentives enough? In a study done by Weissman et al., Israeli students in their fifth year of medical studies were interviewed about their future career choices. The authors found that men were more interested in procedure oriented specialties that allowed for private practice. More women wanted short residencies with few on-calls and limited hours [14]. With this finding in mind, financial incentives alone may only encourage male graduates to prefer the specialties predicted to be in shortage exacerbating existing gender disparities and turning specialties into predominantly male or female fields.

With the increased numbers of women physicians making their decision on specialization at the peak of their of childbearing and child rearing years, incentives to attract women to preferred specialties may have to be more innovative than only direct financial incentives. Israel needs to develop more supportive working environments, flexible and family friendly working hours and training programs, fringe benefits such as close housing and daycare for children that can allow mothers to work - yet be close enough to see their children at times of need. Special mentoring programs could be arranged to prepare and encourage women to join the workforce in the more needed specialties.

### Effects on the quality and style of medical care

Beyond the issue of quantitative planning of the workforce, it is interesting to observe the way that feminization of the profession might change the style and outcomes of medical practice. We did not find data in Israel on this question, but studies abroad have firstly shown that women tend to be better at preventive measures. In a 1993 survey conducted in a Midwestern health plan in the US among a sample of 97,962 patients referred for preventive medicine services, it was found that female patients are more likely to undergo screening including Pap smears and mammograms if they consult a female rather than a male physician, particularly if the physician is an internist or family practitioner [15].

Secondly, other studies have shown that female physicians have different communication skills than males. Roter et al. [16] systematically reviewed and quantified the effect of physician gender on communication during medical visits. They found that female physicians engaged in significantly more ‘patient-centered’ communication, in particular in primary care studies, such as positive talk, psychosocial counseling and emotionally focused talk and had longer visits than their male colleagues.

In another meta-analysis on patient communication to doctors [17], Hall and Roter found that overall patients spoke more to female physicians than to male physicians, disclosed more biomedical and psychosocial information, and made more positive statements. They were also more assertive to female physicians and tended to interrupt them more.

Research needs to be done in Israel as to whether similar differences exist in the style of practice between male and female physicians.

Are these differences good for the patient? As medicine is continuing to recognize the importance of both mental and emotional wellbeing and not just physical health status, the value of these communication skills will also be increasingly appreciated. While some experts view increased patient interruptions as a sign of decreased respect, others contend that they show a greater sense of comfort on the part of the patient engendered by the female physician. A recent study of patient satisfaction [18] has shown that female patients, in particular, appreciated the more caring attitude of female physicians.

The OECD is working on developing a quality indicator measuring patient experience [19] based on overall patient satisfaction with explanations by the doctor, opportunities to ask questions and involvement in the treatment. It would be interesting to study the influence of gender of the physician on outcomes of this indicator, in Israel. Would the reported improved communication with female physicians [15,16] influence the outcomes in Israel, too?

In the final analysis, Phillips and Austin [20] have suggested that although the increase in female physicians might lead to fewer working hours, the quality of patient oriented care they provide, particularly in the primary care specialties that they favor, might overall be beneficial to population health, since research has shown that strong primary care systems can lower mortality outcomes and could positively affect morbidity, too.

## Conclusion

There has been an increase in female physicians in recent years in most specialties, which will probably level off in the future. The changing gender composition of physicians must be taken into consideration when planning for future medical personnel training and licensing especially in light of the shortage of physicians. The different gender needs and preferences should be addressed when restructuring the incentive system.

The difference in work hours and culture of practices dominated by females may also affect medical outcomes but it is to be hoped that a lowering in quantity may be compensated for by increased quality of care, and efficiency. A strong primary care system has been shown to benefit a wide range of health outcomes, so if the current trend of women preferring the primary care specialties continues, and their increasing numbers strengthen primary care it could facilitate improvements in overall health.

Future studies are needed to determine whether indeed this will be the case.

## Competing interests

The authors declare that they have no competing interests.

## Authors’ contributions

MA conceived the analysis. NG analyzed the data, drafted the method and results and edited the paper. ZH directed the analysis and edited the paper. YA and OT researched and drafted the background and discussion. All authors read and approved the final manuscript.

## Authors’ information

Nehama F. Goldberger, Yael Applbaum and Myriam Aburbeh are researchers in health statistics at the Health Information Division of the Ministry of Health. Ziona Haklai is Director of the Health Information Division of the Ministry of Health. Orna Tal is Deputy Director of Assaf Harofeh Medical Center.

## Supplementary Material

Additional file 1 Multiple logistic regression analysis. Click here for file
