# Supplementary material for: Female physicians: trends and likely impacts on healthcare in Israel
Source: Isr J Health Policy Res. 2013 Sep 17;2:37. doi: 10.1186/2045-4015-2-37 (PMC3851286; doi:10.1186/2045-4015-2-37)
Supplement: Additional file 1 — Multiple logistic regression analysis. [file 2045-4015-2-37-S1.pdf]

Additional file 1 – Multiple logistic regression analysis

We also ran a multiple logistic regression model to show the contributory effects of license year and academic origin to the likelihood of physicians aged under 65 in 2011, being female. Table 1 shows the odds ratios for all physicians, all specialists, and the largest specialist groups. They reflect similar trends to those described in the above article.

TABLE 1 HERE

Odds ratios have steadily increased for all physicians and all specialists with license years, reaching 2.49 (CI 2.24-2.77) for all physicians for 2007-2009 licenses compared to those before 1990, and 2.86 (CI 2.50-3.28), correspondingly, for all specialists adjusting for academic origin. However, in the most recent period, 2010-2011, odds ratios were somewhat lower than the peak, particularly for specialists, perhaps showing that the trend towards feminization has slowed down. Similarly, there is a significantly greater chance of female physicians among immigrants compared to Israeli graduates, after adjusting for license year, (OR = 1.59, CI = 1.50-1.68 for all physicians, OR = 1.41, CI =1.31-1.52 for all specialists). However, Israeli-born who went to study abroad had significantly lower chances of being females than those who studied in Israel, OR = 0.29 (CI = 0.26-0.32) for all physicians and 0.31 (CI = 0.26-0.37) for all specialists.

The increasing trend of feminization with license year is strongly shown for specialties of gynecology, pediatrics and family medicine, with odd ratios

reaching 12.37 (CI = 6.55-23.37) for gynecology, 4.21 (CI = 2.84-6.22) for pediatrics and 3.19 (CI = 1.98-5.14) for 2010-2011 license compared to those before 1990. But the odds ratios for the similar trend in internal medicine also significantly high, peaked earlier, for license years 2007-2009 compared to before 1990 (OR = 5.29, CI = 3.72-7.51) and for general surgery and oncology the peak was in 2004-2006. Psychiatry had significantly high odds ratios only for license years 2004-2006 and 2007-2009 compared to before 1990.

Immigrants specialists who graduated abroad had a significantly greater chance of being female compared to Israeli graduates in all the above mentioned specialties, the highest odds ratio being for internal medicine (OR= 1.86 CI 1.57-2.21) but with the exception of general surgery, for which they had a much lower chance (OR = 0.19 CI=0.10-0.37). However, Israeli-born who went to study abroad had significantly lower chances of females than those who studied in Israel in these specialties, except psychiatry.

Results for orthopedics and anesthesiology were not significant.

We see from the logistic regression the trend for increasing female physicians with license year, but which appears to have peaked for all physicians and all specialists and certain specialties such as internal medicine and oncology. Immigrant physicians are more likely than Israeli graduates to be females, while controlling for license year, while Israeli born who graduated abroad are less likely.

Table 1

Odds ratios for logistic regression models giving likelihood of a physician being female for licensed physicians/specialists in 2011 aged under 65

Factors	Effect/group	All physicians	All specialists	Pediatrics	Family medicine	Gynecology	Internal medicine	Psychiatry	General surgery	Oncology
Year of license	1990-1994 vs Before 1990	1.71**	1.28**	1.30	1.28	1.12	2.29**	0.70	1.11	4.17*
	1995-1999 vs Before 1990	2.04**	1.71**	1.84**	2.53**	2.38*	3.34**	0.95	3.06*	5.77*
	2000-2003 vs Before 1990	2.12**	2.14**	2.69**	2.47**	3.79**	4.08**	1.42	3.77*	3.79*
	2004-2006 vs Before 1990	2.23**	2.54**	3.26**	2.50**	4.51**	4.88**	2.53**	6.88**	9.90**
	2007-2009 vs Before 1990	2.49**	2.86**	3.07**	2.59**	9.92**	5.29**	1.92*	5.92*	3.52
	2010-2011 vs Before 1990	2.47**	2.47**	4.21**	3.19**	12.37**	4.25**	1.55	4.41*	3.63
Academic origin	Israeli-born studied abroad[2] vs Israel[1]	0.29**	0.31**	0.19**	0.17**	0.35*	0.28**	0.71	0.17*	0.27
	Immigrants[3] vs Israel[1]	1.59**	1.41**	1.61**	1.27*	1.69*	1.86**	1.55*	0.19**	1.61
C Statistic		0.65	0.64	0.69	0.68	0.74	0.69	0.63	0.74	0.71
Total N		26043	13766	1996	1577	1045	2690	916	582	161

P value: * <0.05 ** < 0.001. Confidence intervals are available from the authors. The results for orthopedics and anesthesiology are not shown, and were not significant.

[1] Graduated in Israel.

[2] Israeli-born who graduated abroad.

[3] Immigrants who graduated abroad.
